# Association between weight-adjusted waist index and kidney stones: a propensity score matching study

**DOI:** 10.3389/fendo.2024.1266761

**Published:** 2024-08-30

**Authors:** Di Chen, Yurun Xie, Quanhai Luo, Wenji Fan, Gang Liu

**Affiliations:** ^1^ Department of Urology, Reproductive Hospital of Guangxi Zhuang Autonomous Region, Naning, China; ^2^ Graduate School, Guangxi Medical University, Nanning, China; ^3^ The key Laboratory of Clinical Diagnosis and Treatment Research of High Incidence Diseases in Guangxi, Department of Urology, The Affiliated Hospital of Youjiang Medical University for Nationalities, Baise, Guangxi, China; ^4^ The Department of Urology, The Second People’s Hospital of Nanning, Naning, China

**Keywords:** weight-adjusted waist index, obesity, kidney stone, National Health and Nutrition Examination Survey, association

## Abstract

**Objective:**

This study aimed to investigate the association between weight-adjusted waist index (WWI), a novel adiposity index, and kidney stone (KS).

**Methods:**

Data were obtained from the National Health and Nutrition Examination Survey 2007–2018. According to the history of KS, participants were divided into the non-stone group and the stone group. Weighted multivariable logistic regression analyses were used to evaluate the correlation between WWI and KS in unadjusted, partially adjusted, and all-adjusted models. A restricted cubic spline (RCS) analysis assessed the association between continuous WWI and KS risk and obtained the risk function inflection point. Then, subgroup analysis based on the risk function inflection point was conducted to dissect the association in specific subgroups. In addition, the above analyst methods were repeatedly performed in populations after propensity score matching (PSM). The receiver operating characteristic (ROC) curve was applied to compare the ability to predict KS occurrence among WWI, visceral adiposity index (VAI), and body mass index (BMI).

**Results:**

Weighted multivariable logistic regression analyses found a positive association between continuous WWI and KS risk in the all-adjusted model [odds ratio (OR) = 1.03; 95% confidence interval (CI), 1.02–1.04; P < 0.001]. In further analysis, the Q4 WWI group was linked to the highest KS risk when compared to the Q1–Q3 group (OR = 1.06; 95% CI, 1.05–1.08, P < 0.001). RCS analysis found a linear significant correlation between continuous WWI and KS risk, and the risk function inflection point is 11.08 cm/√kg. Subgroup analysis confirmed that WWI was associated with KS risk in different groups. After PSM, increased WWI was still related to a high risk of KS. Moreover, the ROC curve demonstrated that WWI has a higher predictive ability of KS occurrence than VAI and BMI (area under curve, 0.612 vs. 0.581 vs. 0.569).

**Conclusion:**

In the US adult population, elevated WWI value was associated with an increased risk of KS. Furthermore, WWI was a better predictor of KS occurrence than VAI and BMI.

## Introduction

Kidney stone (KS) is a common and serious urology disease affecting human health worldwide. In the United States, KS affects 1 in 11 people, and the prevalence reaches up to 11% (1). Additionally, the incidence of KS showed a rising trend and had a high recurrence rate of 50% within ten years (2). Acute pain by urinary obstruction is one of the most common causes of emergency admission in patients with KS. Urinary obstruction can also impair kidney function, leading to renal atrophy or even renal failure. According to statistics, renal colic commonly caused by KS results in 2 million emergency department visits and costs more than $10 billion per year in the United States (3). Therefore, it is important to identify high-risk KS individuals via a simple and effective diagnostic index.

Obesity is acknowledged to be an increasing threat to global public health, which has been previously linked to cardio-cerebrovascular disease, metabolic diseases, etc. Additionally, obesity is closely related to the development of KS. Obligado et al. performed a review and illustrated that weight is associated with urinary pH values, which are linked to uric acid nephrolithiasis formation (4). Furthermore, excessive nutrition intake increases the transportation of calcium, oxalate, and uric acid, all of which increase the risk of KS (5). The body mass index (BMI) is the most commonly used indicator of obesity, and KS has been positively correlated with BMI. However, BMI has some limitations because it cannot differentiate fat and lean mass. Waist circumference, a measure of body fat distribution, is commonly used to construct modified indicators in evaluating obesity. The visceral adiposity index (VAI)—a novel body fat index made of high-density lipoprotein cholesterol levels, BMI, waist circumference, and triglyceride—was also proven to be positively associated with KS (6). Although VAI has a complex calculation method, widespread use is limited. In 2018, Park et al. proposed the weight-adjusted waist index (WWI), a simple and novel parameter for accessing obesity (7). The WWI, based on waist circumference and weight, was proven to be more sensibility in predicting the prognostic outcome of cardiovascular disease when compared to BMI. Previous studies reported that elevated WWI was associated with the occurrence of many disorders. However, more research explored the correlation between WWI and KS.

The purpose of our study was to use the National Health and Nutrition Examination Survey (NHANES) to explore the association between WWI and KS risk based on a propensity score matching (PSM) approach, which can minimize the differences between different participant groups. We hypothesized that participants with a higher WWI would show a higher risk of KS compared to those with a lower WWI.

## Materials and methods

### Data extraction and screening

The NHANES database contains the following data modules: demographic data module, dietary data module, physical examination data module, questionnaire data module, and laboratory data module. All participants were assigned a unique sequence number, which can identify participants in different modules. In addition, each checked item has a unique code in the NHANES. According to the same code, different cycles of NHANES data can be combined to increase the sample number.

The present study included data from 2007–2018 NHANES: 2007–2008, 2009–2010, 2011–2012, 2013–2014, 2015–2016, and 2017–2018 cycle. The demographic data module contains participant demographic information, including age, gender, race, education, household income–to–poverty ratio (PIR), and marital status. Physical examination has a common physical examination index, including waist circumference and weight. The laboratory data module included serum calcium, serum total cholesterol, and serum creatinine. The questionnaire data module contains questionnaire information, including smoking, physical activity, and history of hypertension, diabetes, asthma, and kidney stones. The inclusion criteria were as follows (1): available kidney stone data and (2) available waist circumference and weight data. The following were the exclusion criteria (1): the information on variables including education, marital status, smoking, and physical activity was “missing,” “refused,” and “don’t know” (2); the information on variables including hypertension, diabetes, and asthma was “missing” and “don’t know”; and (3) PIR information was “missing.”

### Outcome and exposure variables

The exposure variable was WWI, calculated as waist circumference (cm) divided by the square root of weight (kg). Waist circumference was measured at the level of the iliac crest in the standing position. A digital weight scale was used to measure participants’ weight while they wore disposable shirts, pants, and slippers as part of the MEC examination gown.

The outcome variable was whether the participant had a KS history. The questionnaire data module contains kidney condition items recorded related questionnaire. Participants need to answer “Yes” or “No” to the question “Ever had kidney stones?” If participants responded “Yes,” they were categorized as the stone group. Otherwise, they were placed in the non-stone group. Due to KS data being limited in 2007–2018, the corresponding cycles of NHANES were included.

### Covariates

In addition to outcome and exposure variables, the remaining variables were covariates. Some of these covariates were treated as categorical variables. Age was categorized into 20–39, 40–59, and 60 years of age or older. Race was categorized into Mexican American, other Hispanic, non-Hispanic White, non-Hispanic Black, and other race including multi-racial. Education level was classified as less than high school, high school, and college or above. Marital status recoded into two groups: married or living with partner, or living alone (windowed, divorced, separated, and never married). PIR was categorized into lower than 1.5, 1.5–3.5, and over 3.5. For smoking; participants were classified as a non-smoker (smoked fewer than 100 cigarettes in a lifetime), former smoker (smoked at least 100 cigarettes in a lifetime but not currently smoking), and current smoker (smoked at least 100 cigarettes in a lifetime and currently smoking). Physical activity was further categorized into moderate or vigorous physical activity according to the questionnaire “Does your work involve vigorous-intensity activity or moderate-intensity activity?” Asthma, hypertension, and diabetes were classified on the basis of the response to the question, “Ever been told you have asthma/high blood pressure/diabetes?” Additionally, serum cholesterol, serum calcium, and serum creatinine were used as continuous covariates. Peripheral venous blood samples were collected without fasting. Serum creatinine and calcium were measured using a Beckman UniCel® DxC800 Synchron, and serum cholesterol was measured using a Beckman Synchron LX20.

### Statistical analysis

Analyses used survey methods for complex sampling designs with appropriate strata, primary sampling units, and sampling weights. Because some of the variables were measured at the mobile examination center (MEC), the MEC examination weights recorded “WTMEC2YR” were used for all analyses. Continuous variables were expressed as means and 95% confidence intervals (95% CI), whereas categorical variables were represented as the number of cases (percentage). The Wilcoxon rank-sum test for complex survey samples and the chi-square test were used to compare the two groups. In addition, WWI was divided equally into four subgroups (Q1–Q4) according to the quartile level from low to high.

Multivariable logistic regression was used to analyze the association between WWI and KS risk in three models. Molde 1: No covariates were adjusted. Mode 2: Age, gender, race, education, marital status, and PIR were adjusted. Model 3: All covariates were adjusted. When WWI is treated as a continuous variable, restricted cubic splines (RCSs) were explored for potential association between WWI and the odd ratio of KS. Based on the risk function inflection point obtained from the RCS curve, the population was partitioned into two subpopulations that were lower or higher than the risk function inflection point. Furthermore, subgroup analysis stratified by age, gender, race, hypertension, and diabetes was applied to examine the association between WWI and KS risk in two subpopulations. The interaction effect of WWI–KS relation and these subgroups was assessed in model 3. A 1:1 PSM analysis with a 0.05 caliper value was performed to adjust for the effects of age, education level, marital status, PIR, race, smoking, physical activity status, asthma, hypertension, and diabetes. Additionally, a similar method was performed to explore the association between WWI and KS risk in the after-PSM population. Afterward, ROC curves were used to analyze the predictive power of three obesity-related indices, including VAI, BMI, and WWI. Two-tailed P-values less than 0.05 were considered statistically significant. Data analysis was performed with R software version 4.2.2 (http://www.R-project.org). Primary R packages included “haven,” “survey,” “gtsummary,” “matchit,” and “ROCR.”

## Results

### Participant characteristics

**Figure 1:**
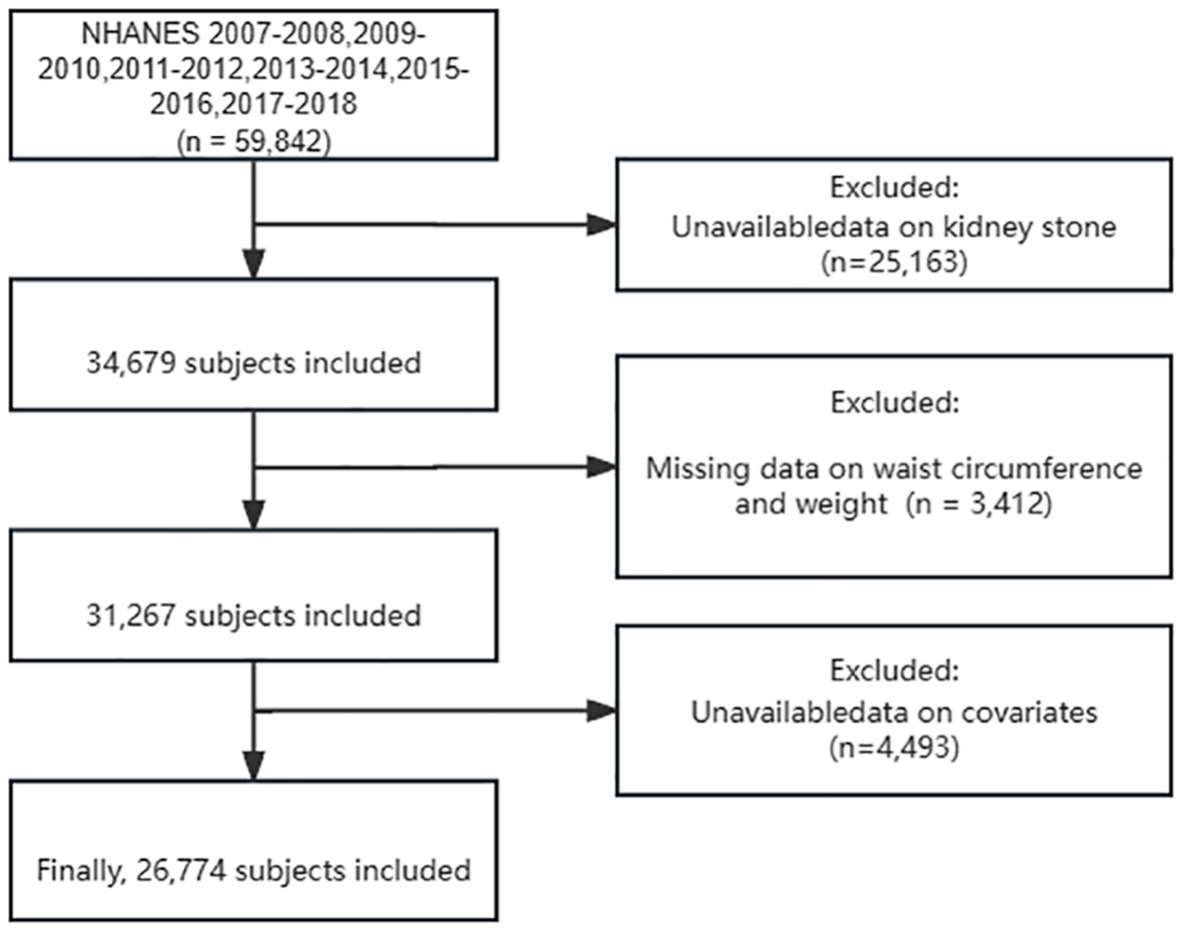
Flow chart selection process.

The NHANES 2007–2018 datasets contained 59,842 participants. After screening and selecting, a total of 26,774 respondents were included in the study. **Figure 1** illustrates the detailed screening flow. Altogether, 2,561 respondents had a self-reported history of KS. The WWI was significantly higher in participants with KS than in those without (11.23 cm/√kg vs. 10.91 cm/√kg, P < 0.001). Participants with KS may be older, male, non-Hispanic white, married or living with partner, former smoker, asthma, hypertension, diabetes, and high serum creatinine (P < 0.05). All the concrete information about the included participants is listed in **Table 1**.

**Table 1:** Baseline characteristics of participants before PSM and after PSM.

Characteristic	Before PSM kidney stone			After PSM kidney stone		
	No (N = 24,213)	Yes (N = 2,561)	P^1^	No (N = 2,560)	Yes (N = 2,560)	P^1^
**Age (years)**	46.0 (32.0, 59.0)	54.0 (41.0, 64.0)	<0.001	54.0 (41.0, 66.0)	54.0 (41.0, 64.0)	0.2
**Sex**			<0.001			0.042
Female	12,586 (52%)	1,120 (45%)		1,222 (48%)	1,119 (45%)	
Male	11,627 (48%)	1,441 (55%)		1,338 (52%)	1,441 (55%)	
**Race**			<0.001			<0.001
Non-Hispanic White	10,034 (67%)	1,417 (77%)		1,299 (76%)	1,416 (77%)	
Non-Hispanic Black	5,094 (11%)	324 (5.5%)		412 (7.4%)	324 (5.6%)	
Mexican American	3,629 (8.6%)	324 (6.0%)		424 (8.1%)	324 (6.0%)	
Other race - including multi-racial	3,024 (8.1%)	220 (6.0%)		195 (4.4%)	220 (6.0%)	
Other Hispanic	2,432 (5.6%)	276 (5.1%)		230 (4.5%)	276 (5.1%)	
**Education level**			0.8			>0.9
Above high school	13,083 (62%)	1,366 (62%)		1,360 (63%)	1,365 (62%)	
Less than high school	5,606 (15%)	631 (15%)		646 (15%)	631 (15%)	
High school	5,524 (23%)	564 (23%)		554 (22%)	564 (23%)	
**Marital status**			<0.001			0.2
Married or living with partner	14,469 (64%)	1,628 (69%)		1,690 (72%)	1,627 (69%)	
Living alone	9,744 (36%)	933 (31%)		870 (28%)	933 (31%)	
**PIR**			0.10			0.7
<1.5	9,063 (26%)	937 (24%)		901 (22%)	937 (24%)	
1.5–3.5	7,785 (31%)	868 (34%)		877 (33%)	867 (34%)	
Over 3.5	7,365 (43%)	756 (43%)		782 (45%)	756 (43%)	
**Smoking**			<0.001			>0.9
Non-smoker	13,635 (56%)	1,251 (50%)		1,264 (50%)	1,251 (50%)	
Former smoker	5,645 (24%)	783 (30%)		773 (30%)	782 (30%)	
Current smoker	4,933 (20%)	527 (20%)		523 (20%)	527 (20%)	
Physical activity status						
**Vigorous**			0.008			0.9
No	19,386 (78%)	2,002 (75%)		1,994 (74%)	2,002 (75%)	
Yes	4,827 (22%)	559 (25%)		566 (26%)	558 (25%)	
**Moderate**			0.9			>0.9
No	15,018 (58%)	1,589 (57%)		1,592 (56%)	1,589 (57%)	
Yes	9,195 (42%)	972 (43%)		968 (44%)	971 (43%)	
**Asthma**			0.002			0.11
No	20,763 (85%)	2,102 (82%)		2,160 (84%)	2,102 (82%)	
Yes	3,450 (15%)	459 (18%)		400 (16%)	458 (18%)	
**Hypertension**			<0.001			0.7
No	15,987 (70%)	1,288 (54%)		1,308 (55%)	1,288 (54%)	
Yes	8,226 (30%)	1,273 (46%)		1,252 (45%)	1,272 (46%)	
**Diabetes**			<0.001			0.030
No	21,389 (92%)	2,007 (82%)		2,073 (85%)	2,007 (82%)	
Yes	2,824 (8.5%)	554 (18%)		487 (15%)	553 (18%)	
**Cholesterol (mg/dL)**	191.00 (165.00, 219.0)	189.00 (164.00, 216.00)	0.014	192.00 (165.00, 220.00)	189.00 (164.00, 216.00)	0.056
**Calcium (mg/dL)**	9.40 (9.20, 9.60)	9.40 (9.10, 9.60)	0.016	9.40 (9.20, 9.60)	9.40 (9.10, 9.60)	0.003
**Creatinine (mg/dL)**	0.84 (0.72, 0.99)	0.89 (0.75, 1.03)	<0.001	0.85 (0.72, 1.00)	0.89 (0.75, 1.03)	0.003
**WWI (continuous)**	10.91 (10.37, 11.49)	11.23 (10.72, 11.78)	<0.001	11.12 (10.61, 11.66)	11.23 (10.72, 11.7)	<0.001
**WWI (categorical)**			<0.001			0.063
Q1	5,515 (26%)	303 (13%)		589 (28%)	494 (23%)	
Q2	5,660 (25%)	492 (22%)		583 (25%)	580 (25%)	
Q3	6,202 (25%)	715 (28%)		667 (25%)	705 (25%)	
Q4	6,836 (24%)	1,051 (36%)		721 (23%)	781 (27%)	

^1^ Chi-squared test with Rao and Scott’s second-order correction; Wilcoxon rank-sum test for complex survey samples.

PSM, propensity score matching; PIR, household income–to–poverty ratio; WWI, weight-adjusted waist index.

### The association between WWI and KS

**Figure 2:**
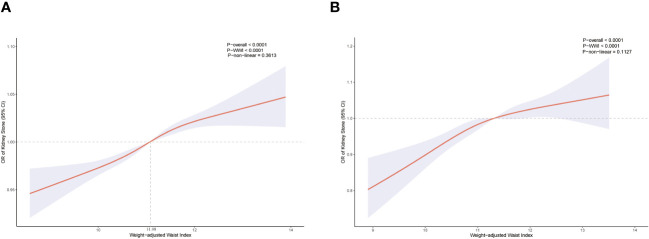
Restricted cubic spline for WWI and KS before propensity score matching **(A)**. Restricted cubic spline for WWI and KS after propensity score matching **(B)**.

In each model, weighted multivariable logistic regression was used to evaluate the association between WWI and KS. **Table 2** shows a significant positive relationship between WWI and KS in all models. The adjusted model (model 3) shows a 3% increase in KS risk for every unit increase in WWI [odds ratio (OR) = 1.03; 95% CI, 1.02–1.04; P < 0.001]. Moreover, the continuous WWI variable was converted to a categorical variable based on WWI quartiles, and correlations between WWI intervals and KS were statistically significant. Model 3 showed a 6% greater risk of KS for respondents in the Q4 quartile (Q2, OR = 1.02; Q3, OR = 1.04). Furthermore, the RCS analysis confirmed a strong linear between continuous WWI and the OR of KS (P-overall < 0.0001, P-WWI < 0.0001, and P-non-linear = 0.3613). As **Figure 2A** shows, 11.08 cm/√kg can be a risk function inflection point, which divides all populations into two subpopulations (<11.08 and ≥11.08).

**Table 2:** Weighted multivariable logistic regression for the association between the WWI and kidney stone risk.

	OR (95% CI), P-value		
	Model 1	Model 2	Model 3
Continuous			
WWI	1.04 (1.04, 1.05) <0.001	1.04 (1.03, 104) <0.001	1.03 (1.02, 1.04) <0.001
Categories			
Q1	Reference	Reference	Reference
Q2	1.04 (1.02, 1.05) <0.001	1.03 (1.01, 1.04) <0.001	1.02 (1.01, 1.04) <0.001
Q3	1.06 (1.05, 1.07) <0.001	1.04 (1.03, 1.06) <0.001	1.04 (1.02, 1.05) <0.001
Q4	1.10 (1.08, 1.11) <0.001	1.08 (1.06, 1.09) <0.001	1.06 (1.05, 1.08) <0.001

WWI, weight-adjusted waist index.

Model 1 was adjusted for no covariates.

Model 2 was adjusted for age, sex, race, education level, marital status, and ratio of family income to poverty.

Model 3 was adjusted for covariates in model 2 + smoking, physical activity status, asthma, hypertension, diabetes, cholesterol, calcium, and creatinine.

### Subgroup analysis

Subgroup analysis of two subpopulations was conducted to examine whether the WWI–KS association was steady in different stratifications. **Table 3** shows that age, sex, hypertension, and diabetes did not affect the independent positive correlation between WWI and KS risk (P < 0.05). The independent positive correlation between WWI and KS may, however, be influenced by race. Except for non-Hispanic white, other races were not found to have a significant association (P > 0.05). Compared to the female group, moreover, the male subgroup had greater KS risk with WWI increased (1.05 vs. 1.02). It seems that subpopulations with WWI ≥11.08 related to higher KS risk than those of WWI <11.08. Detailed analytical results are presented in **Table 3**.

**Table 3:** Subgroup analysis for the relationship between weight-adjusted waist index and kidney stone risk in model 3.

Subgroups	OR (95% CI), P-value		P-value for interaction
	<11.08	≥11.08	
**Age (years)**	Reference		>0.05
20–39	Reference	1.02 (1.00, 1.04) 0.028	
40–59	Reference	1.04 (1.02, 1.06) <0.001	
Over 60	Reference	1.04 (1.02, 1.07) 0.002	
**Sex**			<0.05
Male	Reference	1.05 (1.03, 1.06) <0.001	
Female	Reference	1.02 (1.01, 1.04) <0.001	
**Race**			>0.05
Mexican American	Reference	1.02 (0.99, 1.04) 0.2	
Other Hispanic	Reference	1.02 (0.99, 1.05) 0.3	
Non-Hispanic White	Reference	1.04 (1.03, 1.06) <0.001	
Non-Hispanic Black	Reference	1.02 (1.00, 1.04) 0.055	
Other race - including multi-racial	Reference	1.03 (0.99, 1.07) 0.11	
**Hypertension**			>0.05
Yes	Reference	1.05 (1.02, 1.08) <0.001	
No	Reference	1.03 (1.02, 1.04) <0.001	
**Diabetes**			>0.05
Yes	Reference	1.05 (1.00, 1.11) 0.035	
No	Reference	1.04 (1.02, 1.05) <0.001	

### PSM analysis

In total, 5,120 participants were eligible for analysis after PSM, including 2,560 KS participants and 2,560 non-KS participants. As shown in **Table 1**, there were no statistically significant differences between groups based on age, education level, marital status, smoking, physical activity status, asthma, and hypertension. The results after PSM are shown in **Supplementary Figure 1**. **Table 4** summarizes the results of weight multivariate logistic regression analyses. In model 3, continuous WWI was positively associated with KS risk (OR = 1.07; 95% CI, 1.04–1.10; P < 0.001). Compared to Q1 WWI, Q4 level group have a higher risk for the occurrence of KS in model 3 (OR = 1.14; 95% CI, 1.07–1.20; P < 0.001). Furthermore, the RCS curve appeared to be a linear association between continuous WWI and the OR of KS (P-WWI < 0.0001 and P-non-linear = 0.1127) (**Figure 2B**). Subgroup analysis found that WWI was significantly associated with the risk of KS in the >40 years old, Mexican American, non-Hispanic White, non-Hispanic Black, and non-diabetes subgroups (**Table 5**). Then, an interaction effect was found between diabetes and the WWI–KS risk association (P-value for interaction < 0.05).

**Table 4:** Weighted multivariable logistic regression for the association between the WWI and kidney stone risk after PSM.

	OR (95% CI), P-value		
	Model 1	Model 2	Model 3
Continuous			
WWI	1.05 (1.03, 1.07) <0.001	1.07 (1.05, 1.10) <0.001	1.07 (1.04, 1.10) <0.001
Categories			
Q1	Reference	Reference	Reference
Q2	1.05 (1.00, 1.11) 0.049	1.07 (1.02, 1.13) 0.013	1.07 (1.01, 1.13) 0.013
Q3	1.06 (1.01, 1.11) 0.030	1.09 (1.03, 1.15) 0.003	1.09 (1.03, 1.15) 0.003
Q4	1.10 (1.05, 1.16) <0.001	1.15 (1.09, 1.21) <0.001	1.14 (1.07, 1.20) <0.001

PSM, propensity score matching; WWI, weight-adjusted waist index.

Model 1 was adjusted for no covariates.

Model 2 was adjusted for age, sex, race, education level, marital status, and ratio of family income to poverty.

Model 3 was adjusted for covariates in model 2 + smoking, physical activity status, asthma, hypertension, diabetes, cholesterol, calcium, and creatinine.

**Table 5:** Subgroup analysis for the relationship between weight-adjusted waist index and kidney stone risk after PSM.

Subgroups	OR (95% CI), P-value				P-value for interaction^*^
	Q1	Q2	Q3	Q4	
**Age (years)**	Reference				>0.05
20–39	Reference	1.02 (0.92, 1.12) 0.7	1.00 (0.89, 1.12) >0.9	1.07 (0.93, 1.22) 0.3	
40–59	Reference	1.07 (0.96, 1.19) 0.2	1.08 (0.99, 1.18) 0.086	1.17 (1.06, 1.28) 0.002	
Over 60	Reference	1.12 (1.01, 1.23) 0.03	1.13 (1.02, 1.25) 0.019	1.17 (1.06, 1.28) 0.002	
**Sex**					>0.05
Male	Reference	1.06 (0.99, 1.13) 0.10	1.11 (1.03, 1.21) 0.011	1.14 (1.04, 1.24) 0.005	
Female	Reference	1.07 (0.99, 1.17) 0.10	1.03 (0.95, 1.12) 0.5	1.11 (1.03, 1.19) 0.005	
**Race**					>0.05
Mexican American	Reference	1.17 (1.03, 1.33) 0.016	1.12 (0.98, 1.29) 0.082	1.11 (0.97, 1.27) 0.13	
Other Hispanic	Reference	1.06 (0.90, 1.25) 0.5	0.99 (0.84, 1.17) >0.9	1.04 (0.89, 1.22) 0.6	
Non-Hispanic White	Reference	1.06 (0.99, 1.14) 0.075	1.09 (1.02, 1.17) 0.014	1.14 (1.07, 1.23) <0.001	
Non-Hispanic Black	Reference	1.13 (1.00, 1.28) 0.057	1.15 (1.01, 1.31) 0.030	1.12 (0.97, 1.29) 0.13	
Other race - including multi-racial	Reference	1.05 (0.89, 1.23) 0.6	1.10 (0.95, 1.29) 0.2	1.11 (0.91, 1.36) 0.3	
**Hypertension**					>0.05
Yes	Reference	1.07 (0.97, 1.17) 0.2	1.09 (0.99, 1.21) 0.083	1.12 (1.01, 1.24) 0.029	
No	Reference	1.06 (0.98, 1.14) 0.12	1.07 (0.99, 1.15) 0.095	1.15 (1.06, 1.24) <0.001	
**Diabetes**					<0.05
Yes	Reference	0.88 (0.73, 1.07) 0.2	0.89 (0.73, 1.08) 0.2	0.97 (0.81, 1.15) 0.7	
No	Reference	1.08 (1.02, 1.14) 0.007	1.10 (1.04, 1.17) 0.001	1.14 (1.08, 1.21) <0.001	

PSM, propensity score matching.

*Interaction analysis between selected subgroup and model 3.

### The predictive ability of WWI

**Figure 3:**
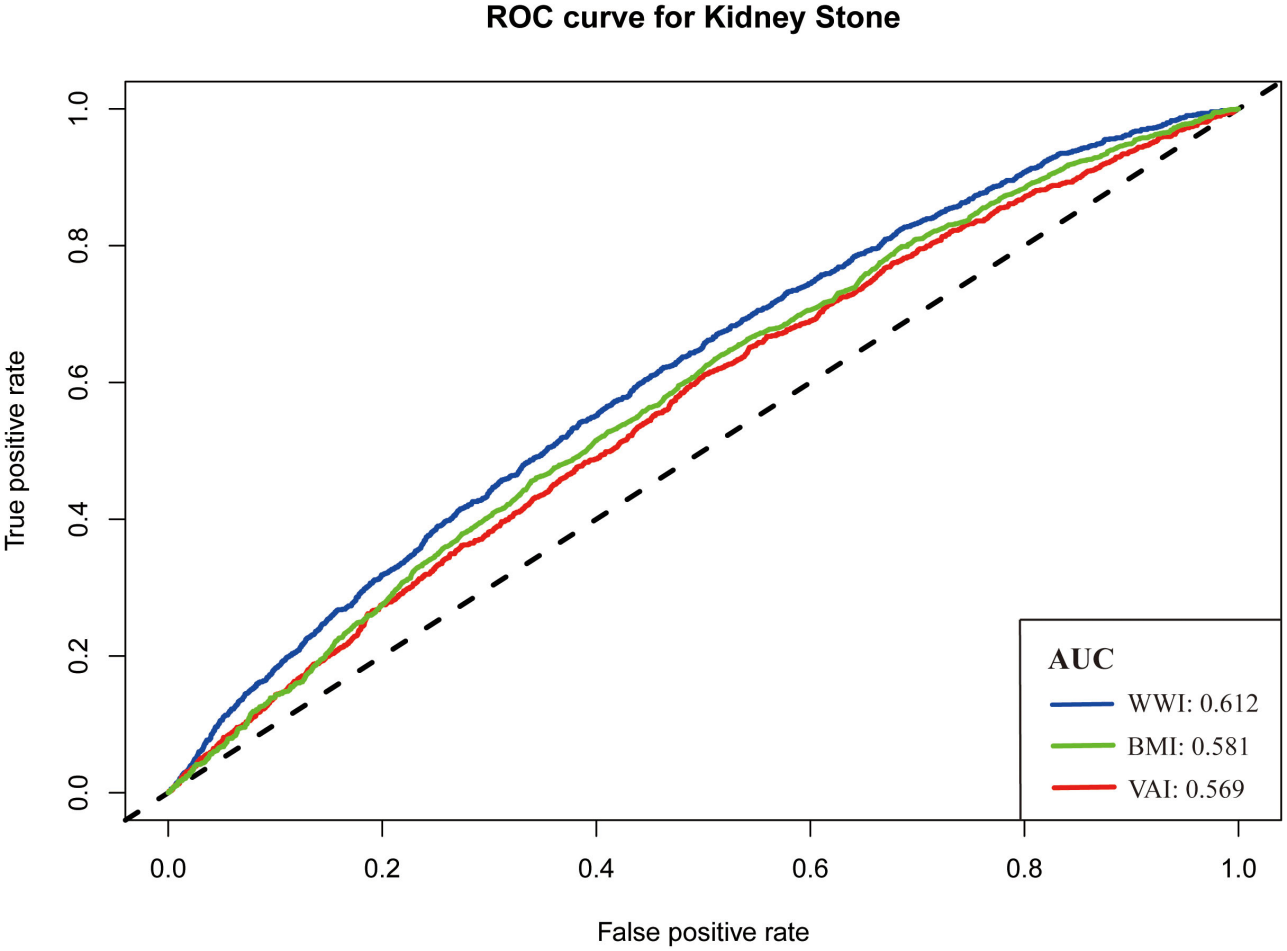
Receiver operating characteristic curve for predicting the occurrence of kidney stone.

The receiver operating characteristic (ROC) curve was used to evaluate the predictive ability of three measurements. In **Figure 3**, the AUC values of KS indicators were shown: WWI, 0.612; BMI, 0.581; and VAI, 0.569. Of the three measurements, WWI had the highest AUC value.

## Discussion

In this study, we included 26,774 participants and found a relationship between WWI and KS risk. In the adjusted all covariates model, Q4 WWI is associated with the highest KS risk. RCS found a significant linear association between continuous WWI and the OR of KS. Subgroup analyses further showed that the positive association was consistent in different subgroups. To better control covariates bias, we performed a 1:1 PSM. Subsequently, the weighted multivariate logistic regression analysis found that an elevated WWI was significantly associated with KS risk. The RCS and subgroup analyses showed consistent results before and after PSM. Additionally, the ROC curve suggested that WWI was superior to VAI and BMI in predicting the occurrence of KS. Our methodology references previously published studies; hence, the results are reliable. Furthermore, we performed a PSM analysis and found a positive relationship, which was not carried out in previous WWI-related studies. Although WWI was reported to correlate with other diseases, our study is the first to explore the correlation between WWI and KS risk by PSM.

As previous studies indicate, BMI was involved in the development and progression of KS. Semins et al. performed a study that included 95,598 patients and found a positive correlation between BMI and KS risk (8). However, the risk of KS had a stable trend in those with a BMI greater than 30 kg/m^2^. Taylor et al. constructed a prospective study that included three large cohorts and found that compared with the male group with BMI of 21–22.9 kg/m^2^, the odd ratio of KS formation was 1.9-fold higher in the male group with BMI of more than or equal to 30 kg/m^2^ (9). Similarly, a meta-analysis of 13 studies by Aune et al. demonstrated a non-linear and positive association between BMI, waist circumference, weight, and KS risk (10). Multiple factors, such as urine composition, hormone level, inflammation, diet, and physical activity, might influence the association. Low urinary pH is a common alteration of urine composition in the obese population. Shavit et al. performed a study that included 2,132 patients with KS and found that, compared to the normal weight group, a higher density of calcium, oxalate, citrate, uric acid, and sodium and a lower pH value of 24-h urine were observed in patients with overweight and obese (11). Similar results were reported by Taylor et al., who found that the risk of KS was higher in patients with obesity, driven mainly by a higher risk of uric acid KS (12). Insulin resistance, common in obese populations, may reduce ammonium synthesis and excretion by activating NH3 interactors at the extremes of the renal tubules, which ultimately mediates the elevation of uric acid and the development of kidney stones (13). Furthermore, renal lipotoxicity in obese populations may cause impaired Na^+^/H^+^ exchange and NH4^+^ secretion in the proximal tubule (14). Obese-related inflammation may increase intestinal oxalate absorption. Consequently, urinary oxalate excretion is elevated (11, 15). It is worth noting that bariatric surgery was associated with oxalate excretion and KS risk. Roux-en-Y gastric bypass surgery, a common bariatric surgery, may lead to hyperoxaluria and KS (16). In contrast, no similar report was found in restrictive bariatric surgery (17). Comprehensively, obesity increases intestinal absorption of oxalate and uric acid, resulting in KS formation.

As an obesity-related indicator, WWI has reported a relationship with many diseases, including albuminuria, abdominal aortic calcification, diabetes, fracture, hyperuricemia, heart failure, hypertension, erectile dysfunction, and asthma. Qin et al. performed a study based on the NHANES and found a non-linear and positive relationship between WWI and abdominal aortic calcification (18). Similarly, Yu et al. found a non-linear correlation between WWI and the risk of asthma but a linear and positive correlation between WWI and the age of asthma attack (19). These diseases have also been reported to be associated with KS. Patel et al. performed a study that included 97 patients with KS and found a high AAC Agatston score in patients with uric acid KS (20). Lee et al. conducted a study that adjusted covariates and found a higher prevalence of asthma in patients with KS and recurrent KS (21). Metabolic syndrome and inflammation might be underlying factors that contribute to the association between KS and asthma. Hypertension and diabetes have been shown to be correlated with high risk of KS. In our study, we also noted a high incidence of diabetes, hypertension, and asthma in the stone group (**Table 1**). However, there is no strong evidence about the correlation between erectile dysfunction and KS. Taylor et al. conducted a prospective study that included 26 years of follow-up and found a relationship between KS and wrist fractures (22). Hypercalciuria may be a common cause of KS and fractures. WWI, as an integrated predictor, may linked to KS by Metabolic syndrome or the above factors. Hence, WWI could reasonably reflect the KS risk.

Other obesity-related indicators, such as abdominal volume index, waist-to-height ratio, BMI, waist circumference, conicity index, waist-to-hip ratio, body roundness index, and triglyceride glucose index, were commonly used to assess the association between obesity and other diseases. Lee et al. evaluated the correlation between these indicators and the risk of KS. They found that only abdominal volume index, BMI, waist-to-height ratio, body roundness index, waist circumference, and waist-to-hip ratio were associated with the prevalence of KS (23). Similarly, Hou et al. performed a study based on the NHANES. They found that visceral obesity index, calculated by high-density lipoprotein cholesterol, waist circumference, BMI, and triglycerides, was associated with KS risk (24). However, only some studies have compared the ability of these indicators to predict KS formation. In our study, we drew a ROC curve to measure the predictive power of BMI, VAI, and WWI. It was found that BMI and VAI had similar predictive ability (AUC, 0.581 vs. 0.569), whereas WWI had better predictive ability (AUC, 0.612). Considering the simplicity of the calculation and the better predictive power, WWI can be used as a new detection tool in the clinic.

Our research also has some limitations. First, the study was conducted using the NHANES database, which is more representative of the US population. Although our results show a positive association between WWI and KS risk, the association may not work in other countries. Second, the data on the KS group came from a positive history of KS. Hence, participants in the KS group may not have suffered from KS at the time of the NHANES interviews. Patients with KS diagnosed by CT imaging or B-scan ultrasonography might be helpful in detecting the association. Third, WWI cannot completely substitute BMI. Our study evaluated the predictive ability of VAI, BMI, and WWI. Compared to BMI, WWI was the body index reported in recent research. The importance of BMI has been well acknowledged by the clinic and live. More studies are needed to verify and confirm the reliability of WWI. Fourth, some potential covariates that may influence KS development, such as food intake and water quality, were not collected due to the lack of related data. A previous study found that the ionic composition of drinking water may be related to the formation of KS (25).

## Conclusion

Our research found that the higher the WWI value, the higher the risk of KS. WWI was inversely linearly associated with KS risk. In addition, WWI was superior to BMI and VAI in predicting KS occurrence. However, more studies were needed to confirm our conclusion further.

## Data Availability

Publicly available datasets were analyzed in this study. This data can be found here: https://www.cdc.gov/nchs/nhanes/index.htm.

## Supplementary material

Supplementary Figure 1 The summary of propensity score matching.
