# Corrigendum: Association between weight-adjusted waist index and kidney stones: a propensity score matching study

**DOI:** 10.3389/fendo.2024.1481393

**Published:** 2024-09-02

**Authors:** Di Chen, Yurun Xie, Quanhai Luo, Wenji Fan, Gang Liu

**Affiliations:** ^1^ Department of Urology, Reproductive Hospital of Guangxi Zhuang Autonomous Region, Naning, China; ^2^ Graduate School, Guangxi Medical University, Nanning, China; ^3^ The key Laboratory of Clinical Diagnosis and Treatment Research of High Incidence Diseases in Guangxi, Department of Urology, The Affiliated Hospital of Youjiang Medical University for Nationalities, Baise, Guangxi, China; ^4^ The Department of Urology, The Second People’s Hospital of Nanning, Naning, China

**Keywords:** weight-adjusted waist index, obesity, kidney stone, National Health and Nutrition Examination Survey, association

In the published article, there was an error in affiliation 3. Instead of “The Department of Urology, The Affiliated Hospital of Youjiang Medical College for Nationalities, Baise, China”, it should be “The key Laboratory of Clinical Diagnosis and Treatment Research of High Incidence Diseases in Guangxi, Department of Urology, The Affiliated Hospital of Youjiang Medical UniveVrsity for Nationalities, Baise, Guangxi, China”.

The authors apologize for this error and state that this does not change the scientific conclusions of the article in any way. The original article has been updated.

