# Supplementary figures and images for: Association between weight-adjusted waist index and kidney stones: a propensity score matching study
Source: Front Endocrinol (Lausanne). 2024 Aug 30;15:1266761. doi: 10.3389/fendo.2024.1266761 (PMC11193331; doi:10.3389/fendo.2024.1266761)

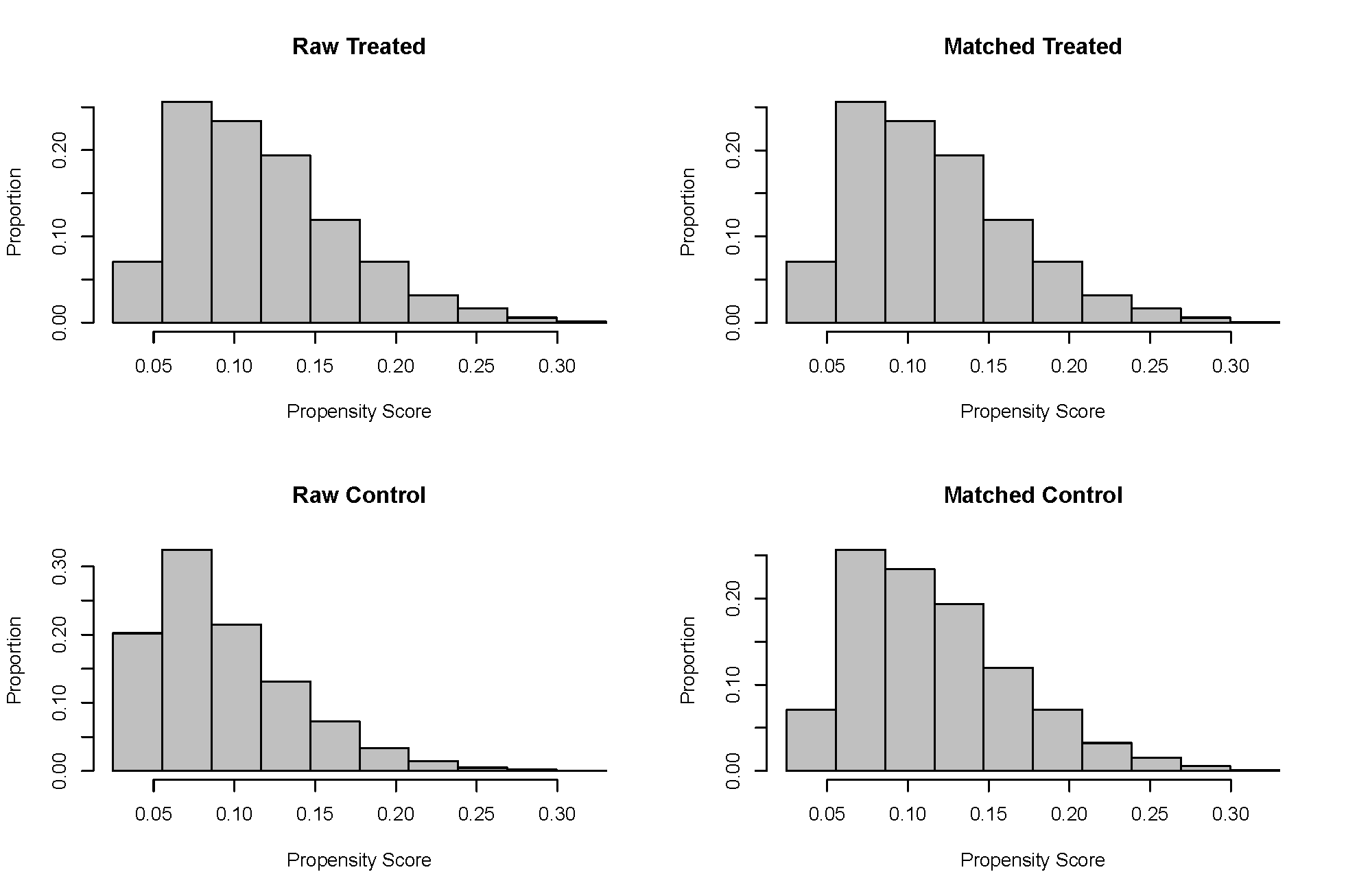

Supplement: Supplementary Figure 1 — The summary of propensity score matching. [file Image1.tiff]
